# Cortical activity increases in speech motor areas as a function of the subjective loudness of inner speech

**DOI:** 10.3389/fnhum.2026.1812507

**Published:** 2026-05-01

**Authors:** Barry H. Cohen, Bin Zhang

**Affiliations:** 1 Department of Applied Psychology, Steinhardt School of Culture, Education, and Human Development, New York University, New York, NY, United States; 2 Mind Tune Psychology, Shenzhen, China

**Keywords:** auditory cortex, fMRI, inner speech, subjective loudness, supplementary motor area

## Abstract

**Introduction:**

Inner speech, sometimes referred to as an inner monologue or silent verbal thinking, is a common mental phenomenon, often experienced as a faint auditory image of words as spoken in one’s own or a more generic voice. Brain-scanning research has shown that inner speech activates many of the same cortical areas responsible for spoken speech. This neural overlap has been leveraged with considerable success recently to decode the content of inner speech from cortical activity using cutting-edge data-analytic methods so that totally paralyzed patients can communicate. The hypothesis underlying the current study is that efforts to create subjectively louder inner speech will be associated with greater neural activity in cortical areas associated with overt speech.

**Methods:**

While they were situated in an MRI scanner, we asked eight participants to repeat simple syllables as either soft or loud inner speech in a randomized order. For comparison purposes, the inner speech trials were followed by participants listening to a recording of the same syllables they made earlier, and a phase during which they repeated those syllables aloud.

**Results:**

As expected, we found significantly greater neural activity in cortical areas associated with speech motor activity during loud as compared to soft inner speech. We also that found greater suppression of neural activity in auditory perception areas was associated with louder inner speech.

**Discussion:**

The results are discussed with respect to their implications for the identification of inner speech in totally paralyzed individuals and the possibility of using neurofeedback to reduce the volume of negative inner speech.

## Introduction

Surveys have found that, for most people, thinking often takes the form of auditory images that resemble the sounds of overt speech (e.g., McCarthy-Jones and Fernyhough, 2011). This subjective phenomenon, informally called “a little voice in the head,” has been variously referred to as subvocalization, an internal monologue (or dialogue), verbal thinking, or covert speech, among other descriptions, but the term that has gained popularity in the last few decades is inner speech. According to Alderson-Day and Fernyhough (2015): “Inner speech can be defined as the subjective experience of language in the absence of overt and audible articulation” (p. 931).

It is important to note that there are considerable individual differences in the vividness and the frequency of the use of inner speech and that there is a small percentage of individuals who claim to not experience inner speech at all (Roebuck and Lupyan, 2020). Moreover, the propensity to experience and use inner speech has quantifiable cognitive consequences. For example, Nedergaard and Lupyan (2024) compared participants whose scores were low or high on an inner speech questionnaire and found that the low inner speech group performed significantly worse on a verbal recall task and were slower and less accurate when deciding whether the words associated with two pictures (e.g., a bone and a cone) rhymed or not.

In modern times, consideration of the subjective quality of inner speech dates back at least to the beginnings of Western psychology in the 1890’s and early 1900’s, with its emphasis on introspection. At that time, motor theories of perception and thinking became popular (see Cohen, 1986, for a historical review). As a result of his own introspection, James (1890/1950) suggested that faint muscle contractions, too small to be detected visually, accompanied inner speech, especially when effort was involved.

In a test of a motor theory of inner speech—that inner speech results from activating the same motor mechanisms as for overt speech, but too slightly to create any sound or visible muscle movement—Jacobson (1932) used an early version of an electromyograph (EMG) to detect faint contractions in the lips and tongue when subjects performed tasks associated with inner speech, like mental counting, or recalling a poem. As expected, the cortical motor activity generated by inner speech produced enough muscle activation to be detected by electrodes placed on the lips or tongue. Using modern EMG equipment, McGuigan and Winstead (1974) confirmed Jacobson’s more qualitative findings by demonstrating significantly higher lip than tongue tension when subjects rehearsed words containing bilabial phonemes (e.g., poppy), and the reverse pattern when rehearsing words with lingual phonemes (e.g., doll).

The development of functional magnetic resonance imaging (fMRI) has made it possible to locate the brain areas that become most active during inner speech. An early fMRI study by Shergill et al. (2001) found that inner speech, compared to a baseline, was associated with “activation in the left inferior frontal/insula region, the left temporo-parietal cortex, right cerebellum and the supplementary motor area” (p. 241). A subsequent review of fMRI studies by Perrone-Bertolotti et al. (2014) confirmed that inner speech is correlated with activation in the left insula, left inferior frontal gyrus (approximately Broca’s area), and the left superior temporal sulcus (auditory cortex). Notably, the activation of the supplementary motor area, which is essential for overt speech generation, has been observed during the articulation of words both overtly and covertly (Alderson-Day and Fernyhough, 2015).

Using fMRI, Tian et al. (2016) compared a condition in which participants were asked to imagine saying (i.e., articulating) a syllable with a condition in which they were asked to imagine hearing that syllable. Both conditions activated speech motor areas, as well as part of Broca’s area, but imagined articulation produced greater activity than imagined hearing in superior temporal (auditory) areas in both hemispheres. The imagined articulation condition corresponds to ordinary inner speech, which includes covert motor activation, whereas imagined hearing is a form of auditory imagery and relies more on memory retrieval. The finding that activation in the auditory cortex accompanies inner speech is consistent with a theory that accounts for the auditory image produced by inner speech in terms of corollary discharge—that is, neural signals sent from a motor area to a sensory area in order to prepare our perception for changes produced by our own motor activity (Ford and Mathalon, 2004; Scott, 2013).

To support that theory, Tian and Poeppel (2010) used magnetoencephalography (MEG), which affords much finer temporal resolution than fMRI, to demonstrate that during inner speech the activation of the speech motor area is earlier than that of auditory cortex and that the time difference is relatively stable. Further evidence for the auditory nature of inner speech has come from a series of perceptual experiments by Tian et al. (2018) in which generating loud inner speech reduced the participant’s subjective loudness of an external sound more than did soft inner speech. That interference between inner speech and external sound is consistent with the finding of Ford et al. (2001) that suppression of auditory ERPs to noise probes occurred while participants engaged in inner speech and confirmed in a study by Jack et al. (2019) who found suppression of an ERP when an externally presented phoneme matched in timing and content to a phoneme participants generated by inner speech.

A very precise measurement of both the location and timing of cortical activity can be obtained from the electrocorticogram (ECoG), which requires inserting microelectrodes directly into exposed cortical areas. Beginning in the early 2010’s, ECoG recordings were being analyzed with the goal of decoding inner speech for the ultimate purpose of facilitating communication in patients with total paralysis, known as the “locked-in syndrome.” Brain-computer interface (BCI) research employing ECoG is presently a highly active area producing excellent discrimination of words expressed as inner speech. Kunz et al. (2025) found specific areas in the motor cortex that allowed highly accurate decoding of inner speech, and Wandelt et al. (2024) were able to significantly discriminate among a small set of words from microelectrodes inserted in the supramarginal gyrus, a key cortical area for language processing.

In order to decode inner speech from participants without implanted electrodes, Dash et al. (2020) analyzed MEG recordings from participants imagining five different phrases (e.g., “I need help”). Using convolutional neural networks for data analysis, discrimination considerably above chance was attained. However, although it is non-invasive, MEG technology is expensive and cumbersome compared to the use of EEG caps. Therefore, recent studies have applied the latest deep machine learning and neural network models to EEG data recorded during inner speech trials and have achieved significant levels of discrimination with a fairly small number of words and/or phonemes (Macias-Macias et al., 2023; Simistira Liwicki et al., 2022).

Although inner speech has been receiving an increasing amount of attention from psychologists and neuroscientists of late, especially with respect to decoding phonemes from EEG data, gaps in the research persist. Despite a thorough literature search, we could not find any study that has systematically investigated the difference in brain activity patterns associated with subjectively soft versus loud inner speech. To address this gap and thereby gain a more thorough understanding of the mechanisms of inner speech, we conducted the fMRI study described herein. Similar to a previous study (Shergill et al., 2006), we asked our participants in the MRI scanner to generate simple consonant/vowel syllables (e.g., “ba”) subvocally in response to a visual cue that flashed briefly at one-second intervals. A separate visual cue informed the participant of which of four syllables to repeat and the loudness to use (soft or loud) for the following 12 s. Note that all the participants found the instructions to repeat syllables as either soft or loud inner speech to be clear and none expressed any difficulty or had any questions with respect to following those instructions. For comparison purposes, the inner speech runs were followed by a listening run, in which the participants listened to a recording they had previously made speaking the same syllables both softly and relatively loudly, and an overt speech run, during which the participants spoke the same syllables out loud.

Given that previous studies have shown that the production of inner speech is related to changes in neural activity in both speech motor and speech perception areas (e.g., Shergill et al., 2002), we focused on these cortical locations. The primary hypotheses of this study were:

H1A: Inner speech will produce activation in some of the same speech motor areas as overt speech.

H1B: Compared to soft inner speech, loud inner speech will produce a higher level of neural activity in the speech motor areas identified with respect to H1A.

Our secondary hypotheses were:

H2A: Compared to listening to soft overt speech, listening to loud overt speech will produce greater activity in auditory perception areas.

H2B: Compared to soft inner speech, loud inner speech will produce a higher level of neural activity in the auditory perception areas identified with respect to H2A.

## Methods

### Participants

Eleven participants were recruited from the Psychology Department at New York University based on their interest in the project and all were paid $45 for completing the 90-min MRI scanning session. Unfortunately, the data from three participants had to be discarded due to technical issues. The eight participants with usable data were all right-handed (5 males, 3 females; age: M = 30 years, range 20–50 years). All participants were fluent English speakers with no history of neurological disorders or recent drug use, and none withdrew before the end of the study. The New York University MRI screening questionnaire was administered as the criterion for subject exclusion; subjects were excluded if they had any risk factor listed on the questionnaire (see Appendix A). This study was approved by the Institutional Review Board (IRB) at New York University (IRB-FY2016-732).

### Design

The two independent variables in this completely within-subjects study were the subjective volume of inner speech (loud and soft) and the type of syllable (‘ba’, ‘pa’, ‘ga’, and ‘ka’), though the analysis of the syllable variable will not be presented here. The dependent variable was the level of neural activation as measured by the fMRI blood oxygen level dependence (BOLD) signal and averaged across the four syllables at each volume level. For comparison purposes, there were also a listening condition and an overt speech condition. Participants were told to keep their eyes open and to look at the small display screen in the scanner at all times, except for rest periods between runs.

### Experimental task

**Figure 1:**
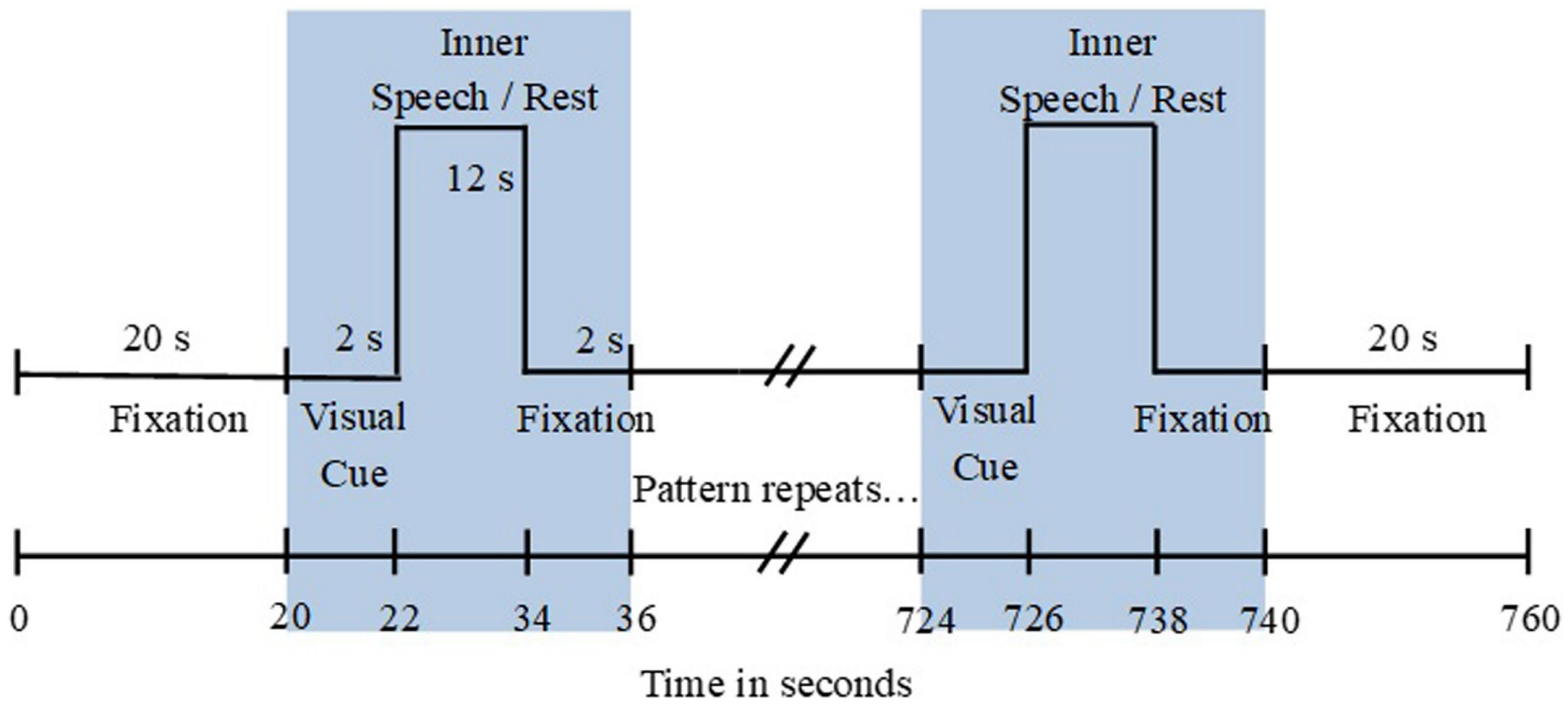
Time sequence of a single run. There were 45 blocks presented between the two 20 s fixations. Each run takes 12 min and 40 s. Participants could take rests between runs so the entire experiment takes approximately 1 h and 30 min.

Each inner speech trial, which we will refer to as a block, began with a two-second visual cue consisting of a volume level and a syllable, with the syllable appearing below the volume level (e.g., Loud/“ba”), followed by a triangle flashing every second for 12 s, and ending with a fixation symbol (a plus sign) for two seconds. Thus, each block lasted a total of 16 s (see Figure 1). The participant was instructed to mentally repeat the cued syllable at the cued volume with each flash of the triangle. In addition to the eight different inner speech blocks, there was a rest block, which used the same timing, except that the 2-s cue was “Rest” and the flashing symbol was an upside-down triangle, and they were instructed to relax and not engage in inner speech while watching the flashing symbol. Thus, there were nine different blocks in all. There were no auditory stimuli during inner speech runs other than the usual noise of the MRI equipment.

A run began with a 20 s fixation period and was followed by 45 blocks (five blocks of each of the nine different blocks) presented in a different quasi-random order for each run, and ending with another 20 s fixation period. Therefore, each run lasted for 12 min and 40 s. For all participants, the first three runs were inner speech runs, followed by a listening run, and an overt speech run. The listening and speaking runs followed the same structure and timing as the inner speech runs, with the participant looking at the appropriate cues and symbols flashing on the screen.

During the listening run, the participants listened in their headphones to the recording they had made before entering the scanner; the syllables were presented in coordination with the flashing triangle and no sound was delivered to the headphones during the rest blocks, when the inverted triangle was flashing, though the sound of the scanner was present. During the overt speech run the participant simply repeated aloud the cued syllable either somewhat loudly or more softly, according to the loudness cue, and stayed silent during the rest blocks. The five runs lasted a minimum of 65 min and a little longer for participants who rested a minute or two between runs.

### Apparatus

The current study was conducted at the New York University’s Center for Brain Imaging (CBI). In this study, a Siemens Allegra 3 T head-only MRI scanner was used. Anatomical images were obtained using an MPRAGE sequence (TE = 3.93 ms, TR = 2000 ms, TI = 900 ms, flip angle = 8 degrees, 176 sagittal slices, 1 × 1 × 1 mm, 256 × 256 matrix in 256 mm FOV). Functional MRI data were acquired in terms of BOLD signal changes using a T2* sensitive echo planar imaging pulse sequence (TE = 30 ms, TR = 2000 ms, 40 axial slices, interleaved, 3 × 3 × 3 mm, 64 × 64 matrix in a 192 × 192 mm FOV). (Note that having the triangle symbol flashing at exactly twice the rate of the TR (1 s vs. 2 s) meant that the syllable production was quasi-synchronized with the TR.) An intercom and model S14 earphones (Sensimetrics Corporation, Woburn, MA) were used for two-way communication between the experimenter and the participant.

### Procedure

When a participant entered the reception room of CBI for their scheduled appointment, they were greeted by a research assistant, who explained the entire procedure, which included that there would be a total of five runs: three inner speech runs; one listening run; and one overt speech run, each lasting approximately 13 min, with rest periods between runs. They were assured that the rest periods had no time limit, they were free to close their eyes, and the following run would not begin until the participant said they were ready. The participant was then asked if they were comfortable signing the informed consent form, and all participants signed.

Next, the participant was given practice repeating each of the four different syllables at two volume levels at a rate of one per second; the experimenter modeled the two volumes that the participants would use to make a recording of their voice for the Listening run. After a few minutes of practice, the participant was left alone in a quiet room to overtly speak the syllables while watching the same cues and with the same timing as the inner speech runs, in order to create the recording for the Listening run.

After the recording was made, participants were brought to the control room of the CBI, where all metal objects were removed from their persons with the aid of a metal detector. Participants then entered the scanner room and were assisted in lying on the MRI table, with foam pads and sheets used to make them comfortable and to limit their movement. After they inserted earplugs, the earphones were placed over their ears. All standard safety measures were implemented per the CBI guidelines.

Once a participant had been situated in the MRI scanner, the experimenter moved to the observation room and informed the participant that the session would begin with a 10-min anatomical brain scan to be used for later analysis, and that there would be no task during that period. After acquiring the anatomical data and making sure the participant was ready to continue, the fMRI session began.

After the session, participants were removed from the scanner, and were asked to complete the Brain Activity Correlates of Inner Speech Debriefing Questionnaire (see Appendix B) about their subjective experiences during the different phases of the scanning session, as well as their daily habit of inner speech. Including all phases of the experiment, the participants spent about 90 min at the CBI.

### Data preprocessing

To remove unwanted variance typically present in fMRI BOLD data, we applied the following preprocessing steps to each participant’s data separately, implemented in SPM8 (Wellcome Department of Cognitive Neurology, Institute of Neurology, London, England), scripted via NeuroElf v1.1.^1^ [Note that the fMRI data for this article was collected and previously analyzed in 2016 as part of an unpublished psychology master’s degree project by co-author BZ.]

First, the structural image was segmented using the Unified Segmentation algorithm, and the skull and surrounding air voxels were masked out (skull stripping). Second, the functional images were slice-time corrected and then realigned and resliced to account for head motion that occurred during each run. Next, a temporal-mean image was computed across all volumes of all runs, the mean image was co-registered to the skull-stripped anatomical image, and all functional images were equally aligned along with the mean image. The anatomical, skull-stripped image was then re-segmented and the derived spatial normalization parameters were used to warp all images (structural and functional) to MNI/ICBM space to account for differences in brain shape across participants. Finally, the resulting normalized functional images were spatially smoothed with an 8-mm full-width-at-half-maximum (FWHM) Gaussian kernel to allow for small differences in activation location across participants and to enable later Gaussian-Random-Fields-based thresholding techniques for whole-brain searches.

### Data analysis

The whole 4D time series of each subject (that is a series of each participant’s three-dimensional volumes) was subjected to a mass-univariate general linear model (GLM) regression, containing predictors separately for each of the nine conditions: loud-ba, soft-ba, loud-pa, soft-pa, loud-ga, soft-ga, loud-ka, soft-ka, rest. Each of these predictors was convolved with the canonical hemodynamic response function (HRF) to account for the delay and sluggishness of the BOLD response relative to the corresponding neural activity (e.g., Logothetis, 2003). In addition, the following predictors were added to account for nuisance variance: temporal filters (removing slow fluctuations below 0.01 Hz) and motion parameters (although displacement of signal location induced by head motion is accounted for during the realignment preprocessing step, the signal as measured by the EPI sequence still contains BOLD unrelated variability due to motion, which can be readily accounted for).

Due to the relatively small number of subjects and the requirements for significance testing using whole-brain, exploratory analyses (i.e., those without a spatial prior, such as a mask or a selection of ROIs), we decided to present only the results of the fixed-effects analyses (random-effects analyses did not yield any significant results). Although the results therefore cannot be used to generalize to the population at large, the effects found in our sample are in line with our theoretical predictions, which adds to the internal validity of our study.

To compare inner speech to rest, after taking the average of all the inner speech blocks (both subjective volumes and all four syllables) and the average of all of the rest blocks, over all three inner speech runs and all eight participants, the difference between the inner speech and rest region estimates was computed. Overt speech versus rest and Listening versus rest were compared in exactly the same way.

To compare soft and loud inner speech, the difference between the soft and loud region estimates was computed as (loud-ba + loud-pa + loud-ga + loud-ga) − (soft-ba + soft-pa + soft-ga + soft-ka). This difference value then represents, for each subject, the degree to which brain activity differs across those two loudness conditions. To assess the whole-brain difference between any two conditions (e.g., inner speech vs. rest) we performed planned fixed-effect paired t tests. The two-tailed significance level was set to a criterion of *p* < 0.001, using a cluster-extent (k voxel) threshold of 25 voxels, as used in other published work on this topic (Tian et al., 2016). Note that the k value was increased to 50 only for display purposes in the brain-image figures to exclude very small clusters, which could be distracting.

## Results

**Figure 2:**
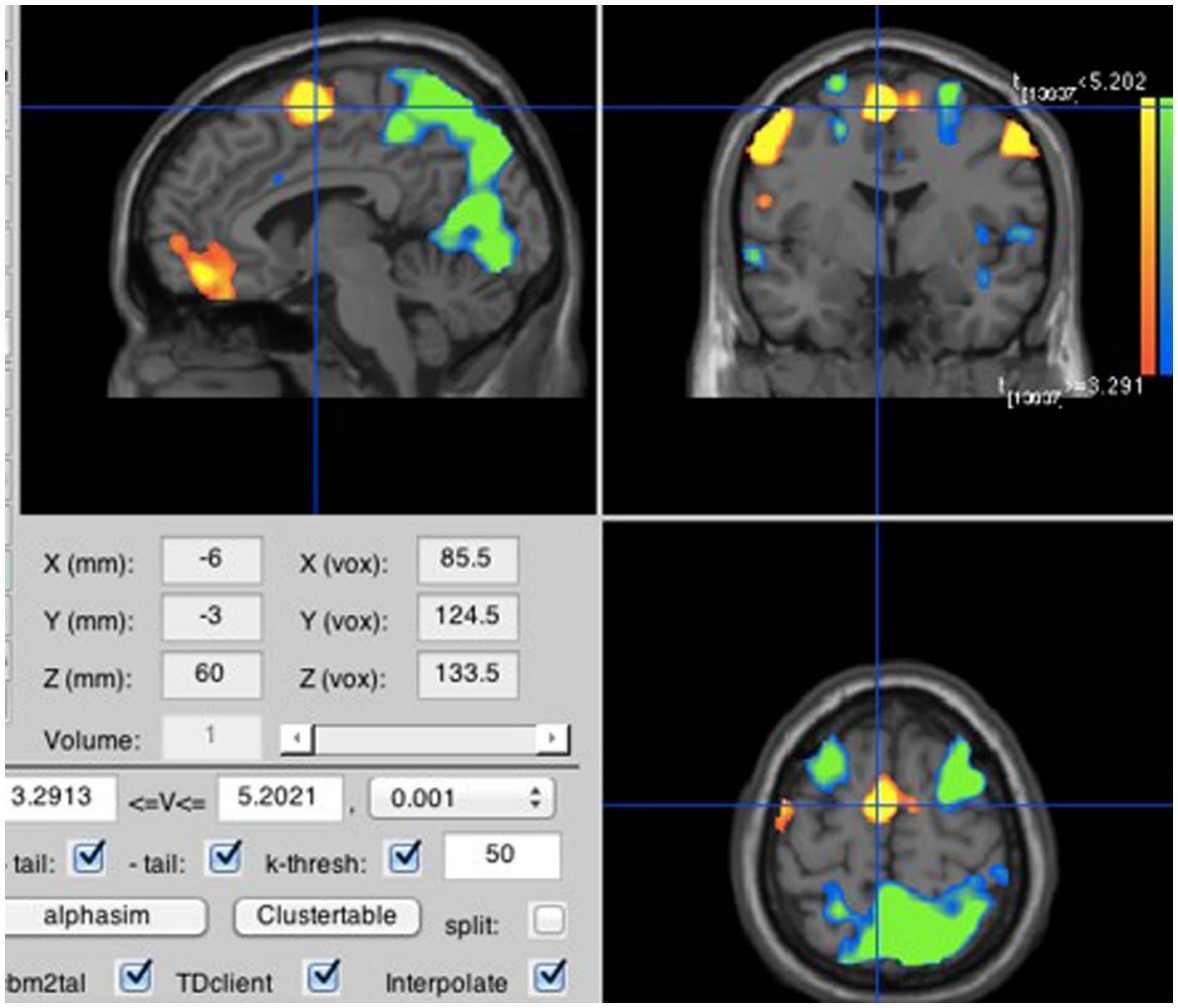
All inner speech compared to rest. Neural activity was stronger for inner speech than rest in the supplementary motor area (MNI coordinates: −6, −3, 60). Note that, in this and the following figures, the yellow/orange bar represents positive *t* values with yellow being the most positive, and the green/blue bar represents negative *t* values with green being the most negative.

**Figure 3:**
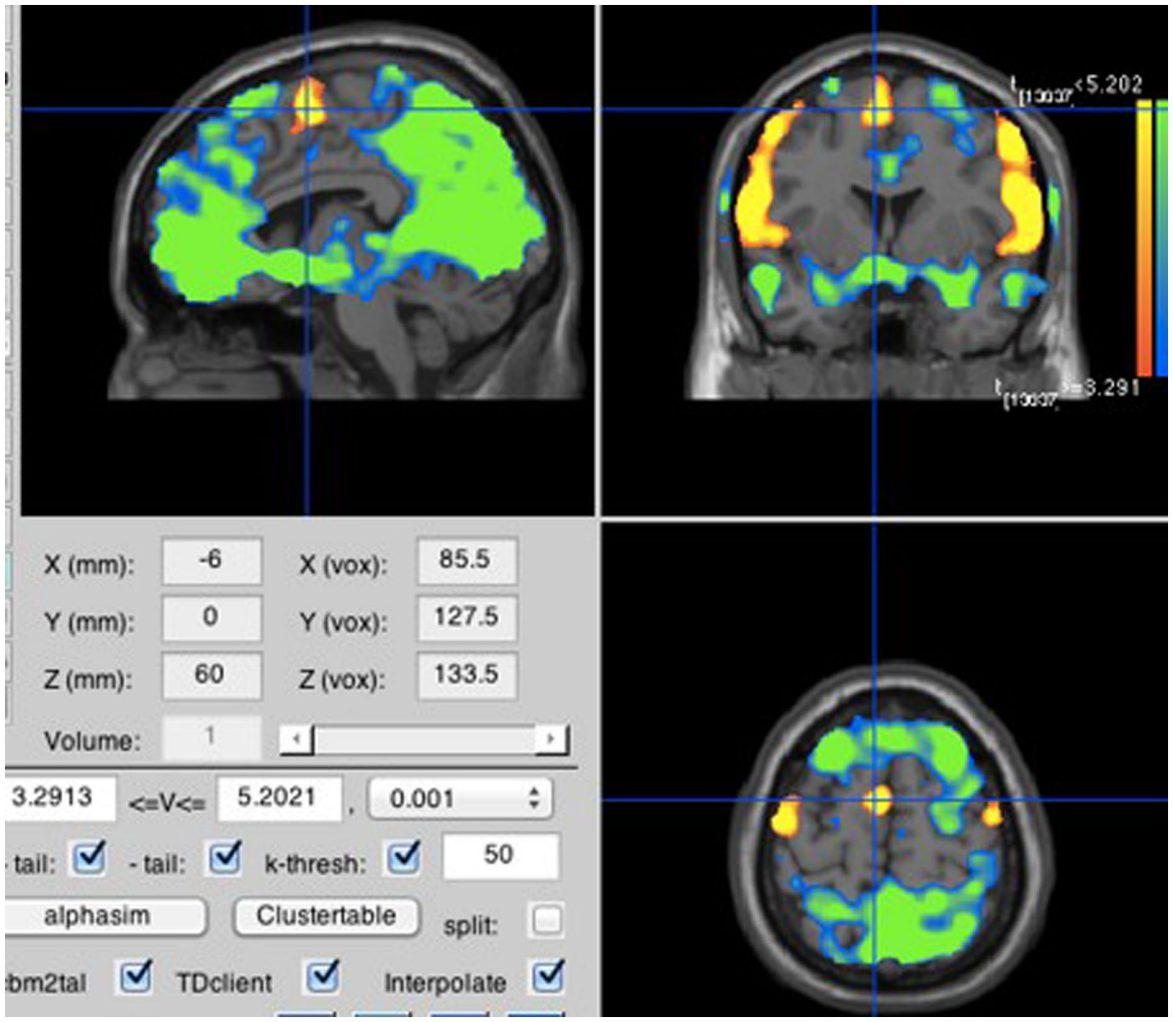
All overt speech compared to rest. Neural activity was stronger for overt speech than rest in the supplementary motor area (MNI coordinates: −6, −3, 60).

A contrast between the averaged inner speech blocks and the averaged rest blocks showed greater activation during inner speech in the supplementary motor area (SMA) within the medial frontal gyrus (see Figure 2). As predicted by H1A, the neural activity generated by inner speech reaches a peak in the same location as the neural activity produced by the averaged overt speech blocks as compared to rest (see Figure 3).

**Figure 4:**
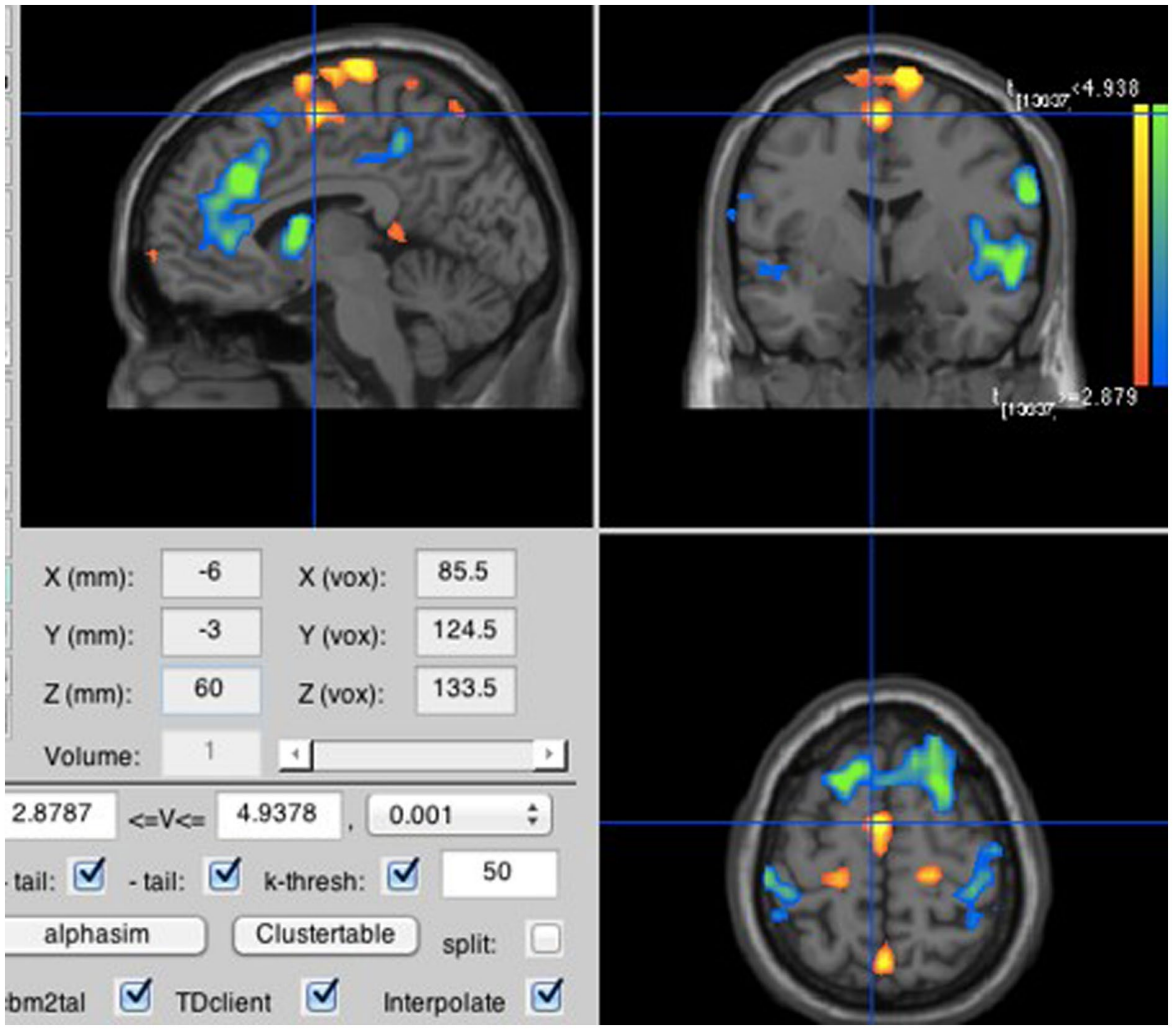
Loud compared to soft inner speech. Neural activity was stronger for loud than soft inner speech in the supplementary motor area (MNI coordinates: −6, −3, 60).

To evaluate H1B, the loud inner speech blocks and the soft inner speech blocks (over all four syllables and all runs) were averaged separately. The contrast between the loud and soft inner speech blocks indicated that loud inner speech is associated with greater activation than soft inner speech in the SMA (Figure 4).

**Figure 5:**
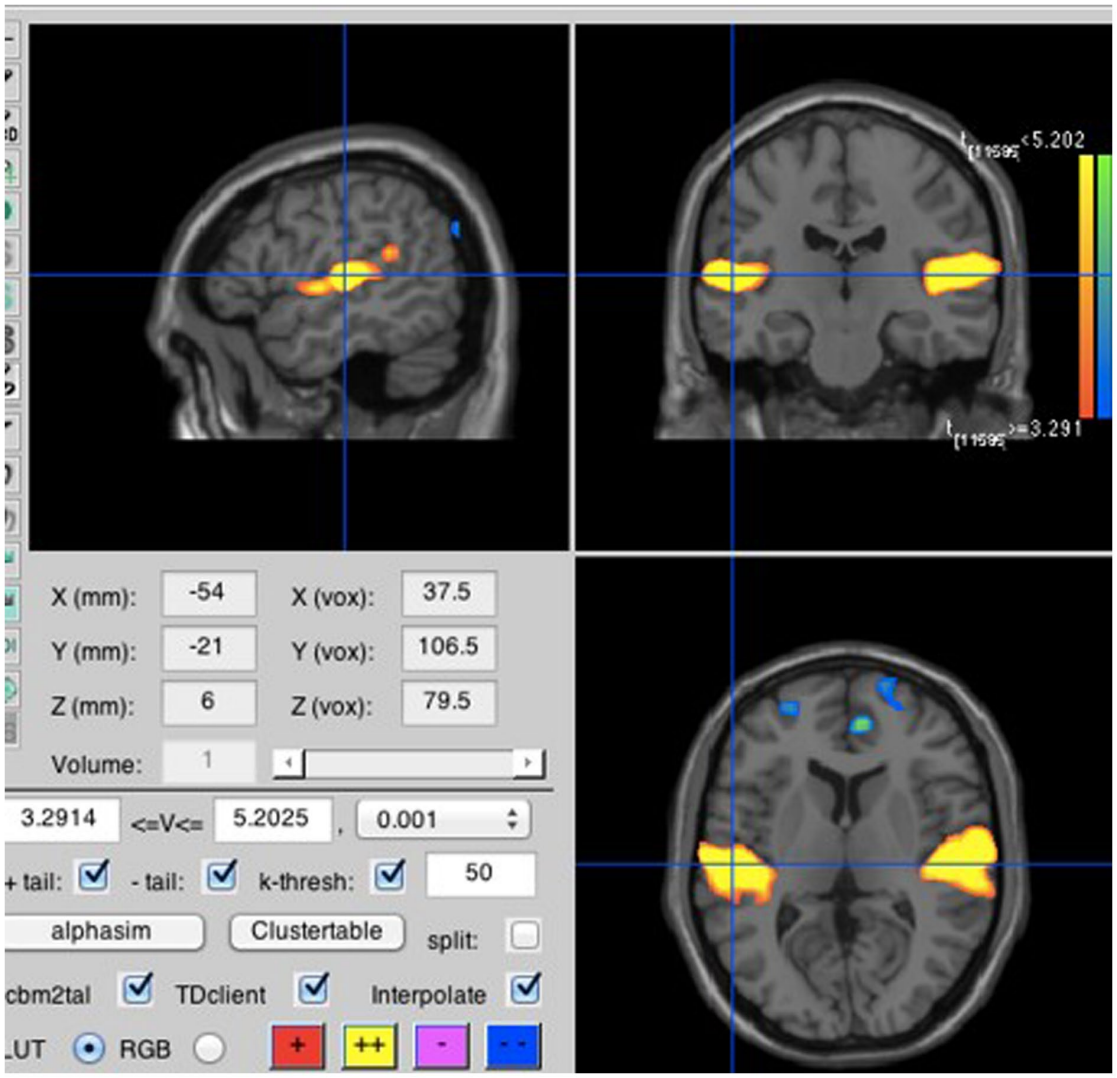
Listening to loud compared to soft recorded speech. Neural activity was stronger for the loud than the soft condition in the lLHG (MNI coordinates: −54, −21, 6).

**Figure 6:**
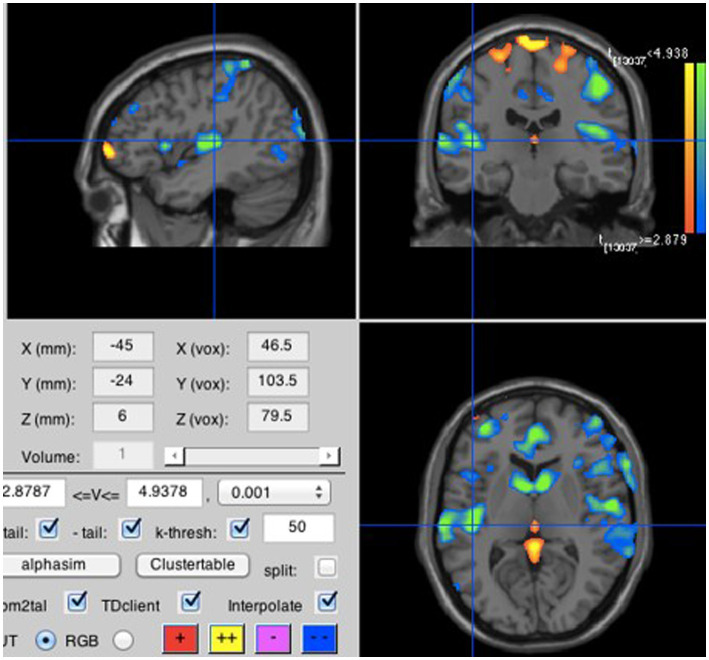
Loud compared to soft inner speech. Loud inner speech was associated with significantly less neural activation than soft inner speech in the lMHG (MNI coordinates are −45, −24, 6).

**Figure 7:**
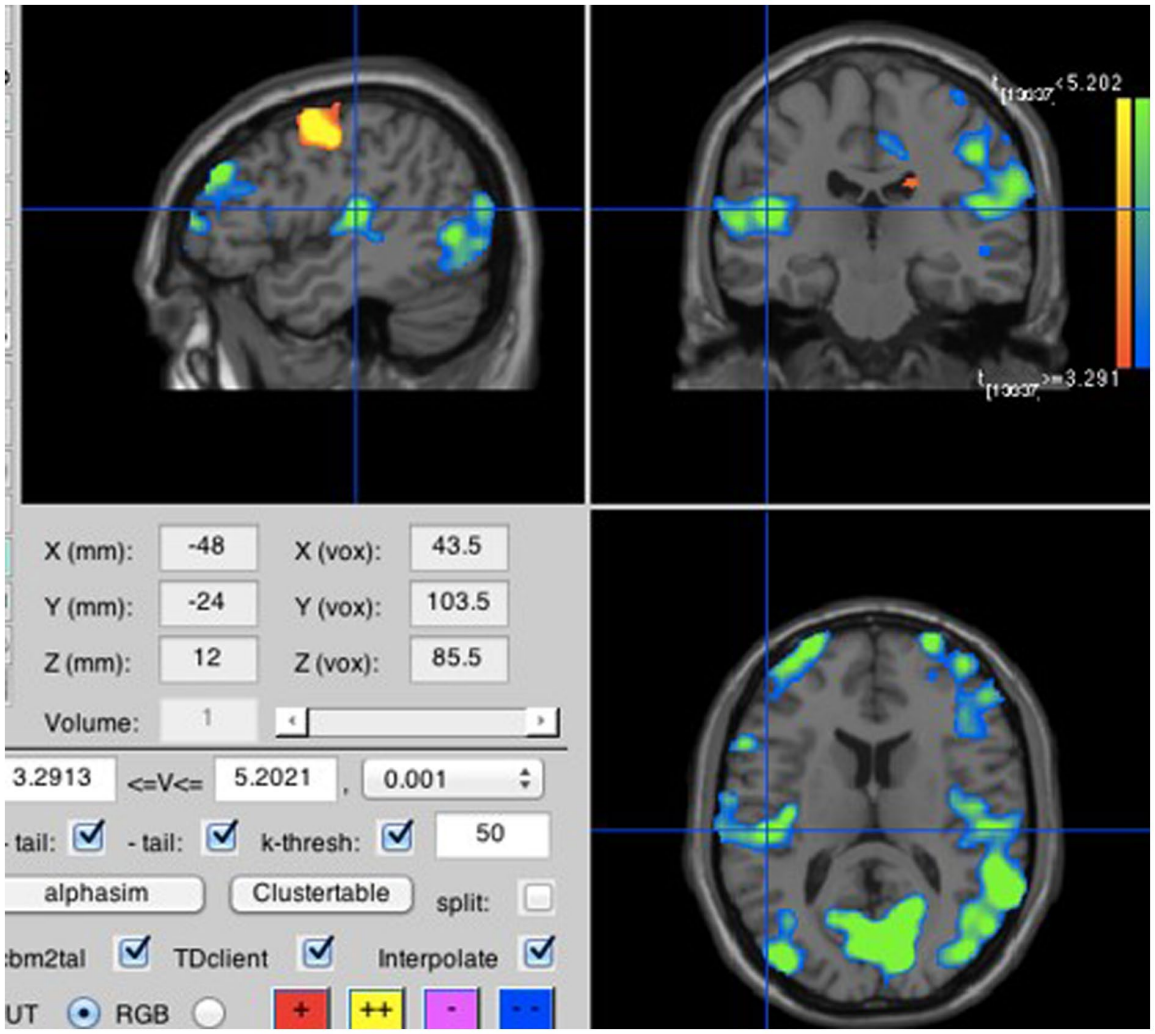
Loud inner speech compared to rest. Neural activity was suppressed relative to rest in the lMHG (MNI coordinates are −48, −24, 12).

To test our secondary hypotheses, we focused on the run during which participants listened to a prior recording of their own voice repeating the four syllables either loudly or softly; the soft listening and loud listening blocks were averaged separately over all four syllables. Consistent with H2A, loud listening led to greater neural activity in the primary auditory perception area; more specifically in the left lateral Heschl’s gyrus (lLHG; Schönwiesner et al., 2007), also known as the transverse temporal gyrus (see Figure 5). In a seemingly paradoxical result, loud inner speech was associated with significantly less activation in the left medial Heschl’s gyrus (lMHG; Schönwiesner et al., 2007; see Figure 6). However, Figure 7 makes it clear that this result is due to loud inner speech significantly suppressing activity in the lMHG as compared to rest, whereas soft inner speech did not produce a noticeable suppression in the same area.

### Debriefing questionnaire

Part I of the questionnaire asked about the amount of spontaneous inner speech participants engaged in while in the scanner waiting for the tasks to begin. None of them responded “Not at all,” and the modal response was “A fair amount.” Part III asked the same question about the rest periods and this time the modal response was “Very little.”

Part II asked two questions about the participants’ subjective experiences while repeating the syllables as inner speech. Responses to the first of these questions revealed that as compared to hearing an image of a generic voice, all of the participants felt that the image was either definitely their own voice or more like their own. The second question asked whether their experience was more like hearing a voice or more like just being aware of the syllables. All but one participant responded “Definitely like hearing a voice” or “More like hearing a voice than not.”

Part IV asked about the frequency of inner speech engaged in by the participants in their daily lives. In this case, one participant responded “Rarely,” whereas the average of the other responses was “Very often.” The responses to Part III suggest that the participants were able to refrain from engaging in inner speech during the rest periods, at least as compared to their degree of engagement at other times.

## Discussion

Consistent with prior research, our results confirmed the first part of our primary hypothesis (H1A). Compared to rest, inner speech was associated with enhanced neural activity, as measured by the BOLD signal, in the supplementary motor area (SMA). This result was consistent with Kunz et al. (2025), who found “representations for attempted speech, inner speech, listening, and silent reading in the same regions of the precentral gyrus …” (p. 4659). Our novel finding was the confirmation of H1B—that is, loud inner speech produced greater neural activity than soft inner speech at the same SMA location that was activated by overt speech. This result clearly implies that our participants had no difficulty modulating the loudness of their inner speech while not producing any sound that could be detected by researchers listening to a microphone placed near the participant’s head in the scanner. That loud inner speech produces greater motor activity than soft inner speech supplies further support for the theory that inner speech is an auditory image generated by corollary discharge directed at speech perception areas in the temporal lobe with the further implication that greater motor activity produces a stronger corollary discharge which, in turn, produces a subjectively louder auditory image.

Consistent with previous research, our results confirmed the first part of our secondary hypothesis (H2A). Compared to rest, listening to relatively loud speech was associated with enhanced neural activity in an area within the lateral portion of the left Heschl’s gyrus. Somewhat unexpectedly, however, engaging in loud inner speech produced less neural activity in the medial portion of the left Heschl’s gyrus (lMHG) than listening to soft inner speech. This difference was not due to soft inner speech producing more activity in lMHG, but rather it was due to loud inner speech producing more suppression of lMHG activity than the negligible amount produced by soft inner speech.

One possible explanation for the suppression of neural activity in the auditory cortex we found for loud inner speech is consistent with research we cited earlier by Ford et al. (2001). Recall that these authors found that, compared to baseline, auditory ERPs to noise probes were significantly suppressed while their control participants engaged in inner speech. Therefore, given the noise generated by the MRI scanner, which is not completely reduced by the earplugs and headphones our participants were wearing, we may be seeing an active suppression of the MRI noise in order to better “hear” the images of inner speech. If that is true, then it is possible that the greater effort involved in loud inner speech was able to produce more suppression than did soft inner speech. This explanation is admittedly speculative and the peak of suppression in Heschl’s gyrus was more medial than the peak of activity for loud versus soft listening to external speech. However, it is not unreasonable to suppose that different locations within Heschl’s gyrus may be activated by the noise of the scanner as compared to the processing of speech sounds.

The debriefing questionnaire confirmed that all but one of our participants engaged in inner speech at least somewhat often in their daily lives and, while repeating syllables mentally in the scanner, all but one participant tended to “hear” what sounded like more like their own voice than not. It was also helpful for our resting baseline that most of the participants reported engaging in inner speech “very little,” during the resting blocks.

### Limitations

Given that the sample size was small and the task being investigated was a novel one, the experiment reported herein can be considered a pilot study in need of replication with a larger and more diverse sample (nearly all participants were graduate psychology students). Because of the small sample size, we did not correct for multiple comparisons. Although none of our results would have survived a correction for multiple comparisons for our whole-brain analysis, note that the results we have presented are consistent with extensive prior research with respect to the cortical locations associated with both overt and inner speech. The consistencies we found in the locations of neural activity among inner speech, overt speech and listening to speech in the present study, as well as consistencies with the previous literature (Alderson-Day and Fernyhough, 2015; Shergill et al., 2001), lend additional credibility to our findings, making them worthy of further exploration.

Another limitation is that we did not, with any precision, control the volume of the soft and loud speech in the overt speech task nor in the making of the recordings for the listening task. In a follow-up study a decibel meter should be used for greater control. Because the loudness of inner speech is purely subjective and cannot be measured independently, a longer period of training and practice with inner speech on the part of the participants might have been helpful.

One of our reviewers alerted us to the fact that fixed-interval (rhythmic) sensory stimulation can induce cortical motor activation, including in the SMA, that is unrelated to our inner speech task, as part of a sensory predictive coding mechanism (Bengtsson et al., 2009). Therefore, we suggest that a future study eliminate this potential confound by replacing the flashing visual stimulus with a static fixation symbol and having the participant mentally repeat the syllables at a previously rehearsed pace.

### Future directions

In addition to replicating our study with a larger, more diverse sample, it would be useful to redo the study using a dense array of EEG electrodes, because that measuring system is less expensive, more portable, and much less noisy than an MRI scanner. However, given the loss in spatial localization associated with EEG as compared to fMRI and, especially, ECoG, it could be helpful to increase the amplitude of the EEG signal during inner speech by coaching participants to engage in somewhat louder inner speech.

With respect to this point, it is relevant to note that Kunz et al. (2025) investigated attempted speech, as well as inner speech in totally paralyzed participants. With respect to speech BCI’s, they noted that: “Current systems require users to attempt to produce speech to the best of their ability (“attempted speech”), which can be tiring and may have inherent speed limitations for paralyzed users” (p. 4659). The results of the present study suggest that it might be helpful for decoding purposes if paralyzed patients were encouraged to produce somewhat louder inner speech rather than attempting to speak. Louder inner speech could be expected to increase the level of relevant neural activity being observed, thus increasing the signal-to-noise ratio. An increase in neural activity could be especially helpful when using less sensitive brain scanning methods, such as EEG.

Another possible application of our findings is that neurofeedback could be given to participants to help them learn how to reduce the subjective volume of their inner speech. This training presents the possibility of teaching those who engage in excessive negative rumination to better control their verbal thinking. At the least, this training could be expected to reduce tension in the speech musculature that spills over from inner speech and, perhaps, reduce general arousal.

## Conclusion

The results of the present study suggest that in order to increase the subjective volume of their inner speech, participants needed to increase their cortical activation in motor areas associated with the production of overt speech—that is, exert more speech-related effort, without making any sound. Conversely, in order to create greater neural activity in speech motor areas that can be picked up more accurately (i.e., with a higher signal-to-noise ratio) by brain scanning devices in future studies, participants would need to be coached to create a subjectively louder auditory image of the words or syllables being targeted. This bit of methodological advice could be helpful to researchers in the rapidly advancing field of speech decoding from brain activity.

## Data Availability

The datasets presented in this study can be found in online repositories. The names of the repository/repositories and accession number(s) can be found at: Open Science Framework: https://osf.io/agre8/files/osfstorage.

## Footnotes

1
https://neuroelf.net/
