# Supplementary material for: Cortical activity increases in speech motor areas as a function of the subjective loudness of inner speech
Source: Front Hum Neurosci. 2026 May 1;20:1812507. doi: 10.3389/fnhum.2026.1812507 (PMC13176268; doi:10.3389/fnhum.2026.1812507)
Supplement: Supplementary file 1 [file Supplementary_File_1.zip › Supplementary Material/Appendix A.PDF]

NYU MRI Center
Subject Screening Form

Name ____ Date _________________

Age ____ Weight ______ Gender ________ Race/Ethnicity (optional) ________________

Principal Investigator _________________________ Study ID __________

**Please indicate if you have any of the following:**	**Yes**	**No**
History of Head Trauma	___	___
Surgical Aneurysm Clips	___	___
Cardiac Pacemaker (even if taken out)	___	___
Prosthetic Heart Valve	___	___
Neurostimulator	___	___
Transdermal Patch (nicotine, nitroglycerin, hormone)	___	___
Implanted Pumps	___	___
Cochlear Implants	___	___
Metal Rods, Plates, Screws	___	___
Previous Surgery	___	___
IUD	___	___
Hearing Aid	___	___
Dentures, Braces, or Non-removable Retainer	___	___
Acupressure beads	___	___
History of Injury to Eye Involving Metal	___	___
Any metal in the body – shrapnel, bullets or buckshot	___	___
**Please answer the following questions:**		
Do you typically experience Claustrophobia?	___	___
Is there any chance you might be Pregnant?	___	___
Are you wearing colored contact lenses?	___	___
Do you have any Tattoos on your face (e.g., eyeliner tattoo), neck, shoulders, or upper back?	___	___
Have you ever worked as Welder or Metal Worker?	___	___

If you answered "**YES**" to any item above, please explain:

All subjects MUST wear either earplugs or headphones during any Imaging.

I have received a copy of the informed consent document for this study (initial here) ______

Signature _________________________________ Date _____________

Witness __________________________________ Date _____________

DO NOT ENTER THE SCAN ROOM WITH ANY OF THESE ITEMS

Credit/Bank Cards	Wallet/Money Clips	Underwire Bra	Pens/Pencils	Coins
Body Piercing	Hairpins/Barrettes	Safety Pins	Glasses	Jewelry
Watch	Keys	Pocket Knife	Wigs/Hairpiece	Belts/Buckles
