# Supplementary material for: Cortical activity increases in speech motor areas as a function of the subjective loudness of inner speech
Source: Front Hum Neurosci. 2026 May 1;20:1812507. doi: 10.3389/fnhum.2026.1812507 (PMC13176268; doi:10.3389/fnhum.2026.1812507)
Supplement: Supplementary file 1 [file Supplementary_File_1.zip › Supplementary Material/Appendix B.pdf]

Appendix B

Brain Activity Correlates of Inner Speech

Debriefing Questionnaire

The purpose of this questionnaire is to obtain descriptions of your subjective experiences during the different parts of your MRI session.

Part I: These questions refer to the period just after you were slid into the scanner, and before the syllable repetition task began.

1. During this period, to what extent were you engaged in spontaneous inner speech (i.e., verbal thinking)? Circle one of the following choices:

Not at all   Very little   A fair amount   Most of the time   Nearly all of the time

2. During this period, what was your mood like? Circle all that apply:

Somewhat fearful   Bored   Somewhat annoyed   Curious   Somewhat tense

3. Please add any relevant comments about your subjective experiences during this phase of the study:

____

____

____

Part II: These questions refer to the period during which you were mentally repeating the syllables.

1. When you were repeating the syllables, to what extent did it seem like you were hearing your own voice versus a generic or neutral voice? Circle one of the following four phrases:

Definitely my own voice   More like my own voice than not

Definitely a generic voice   More like a generic voice than not

2. When you were repeating the syllables, to what extent did it seem like you were *hearing* a voice rather than just being aware of the syllables? Circle one of the following four phrases:

Definitely hearing a voice   More like hearing a voice rather just aware

Definitely just aware of the syllables   More like being just aware than like hearing a voice

3. Please add any relevant comments about your subjective experiences during this phase of the study:

____

____

____

Part III: These questions refer to the period after the syllables task, while you were just resting.

1. During this period, to what extent were you engaged in spontaneous inner speech (i.e., verbal thinking)? Circle one of the following choices:

Not at all   Very little   A fair amount   Most of the time   Nearly all of the time

2. During this period, what was your mood like? Circle all that apply:

Somewhat fearful   Bored   Somewhat annoyed   Curious   Somewhat tense

3. Please add any relevant comments about your subjective experiences during this phase of the study:

____

____

____

Part IV: These questions refer to your use of inner speech in everyday life.

1. During the waking hours of a typical day, how often do you talk to yourself mentally (i.e., “hear,” or be aware of words in your mind, without moving your lips or making any sound)? Circle one of the following choices:

0	1	2	3	4	5
Never	Rarely	Occasionally	Somewhat often	Very often	Almost always

2. How often do your thoughts come to you *not* as distinct words, but more as images, feelings, or vague ideas? Circle all that apply:

0	1	2	3	4	5
Never	Rarely	Occasionally	Somewhat often	Very often	Almost always

Participant's signature: ________________________________ Date: ______________

Thank you very much for participating in this study!
